# The humanitarian-development nexus: humanitarian principles, practice, and pragmatics

**DOI:** 10.1186/s41018-020-00086-0

**Published:** 2020-12-10

**Authors:** Jon Harald Sande Lie

**Affiliations:** Norwegian Institute of International Affairs (NUPI – www.nupi.no), PO Box 7034, St Olavs Plass, 0130 Oslo, Norway

**Keywords:** Development, Humanitarian, Humanitarian–development nexus, Mission creep, Uganda

## Abstract

The humanitarian–development nexus is increasingly being cast as the solution to humanitarian concerns, new and protracted crises, and to manage complex war-to-peace transitions. Despite widely endorsed amongst policymakers, this nexus presents some challenges to those implementing it. Humanitarian action and development assistance represent two distinct discursive and institutional segments of the international system that are hard to juxtapose. Humanitarianism’s apolitical and imminent needs-based approaches building on established humanitarian principles are fundamentally different from the more long-term, political, rights-based approaches of development. As they rub shoulders, as intentionally instigated by the nexus, they affect and challenge each other. These challenges are more acute to the humanitarian domain given the constitutive status of the humanitarian principles, which, when challenged, may cause changes to the humanitarian space and a mission-cum-ethics creep. This article explores the formation and effects of the humanitarian–development nexus as rendered both at the top, amongst policymakers, and from the bottom. The latter explores the discursive transition from conflict to reconstruction in Northern Uganda. Humanitarian organisations’ different response to the transition demonstrate more pragmatic approaches to the humanitarian principles and thus how the nexus itself is also formed bottom up and further exacerbates the mission creep.

## Introduction

The humanitarian–development nexus is a hot topic and has been so for a whilst, creating expectations as well as problems to policymakers and practitioners of humanitarian action and development assistance alike (Eade and Vaux 2007; Hilhorst 2018; IDS 2018)^1^. Basically, the nexus refers to “the transition or overlap between the delivery of humanitarian assistance and the provision of long-term development assistance” (Strand 2020: 104). The nexus’ rationale is fairly straightforward: as the war-to-peace transition is understood in terms of a continuum, one should not compartmentalise but motivate distinct actors to cooperate regardless how the situation is defined. This has, however, proven easier said than done. Not only are the discursive segments of humanitarianism and development distinct to each other, but the segments themselves are characterised by great internal diversity—and these differences become amplified by the nexus itself. The nexus seeks to merge well-established discursive, institutional and attitudinal differences that are hard to reconcile, especially as seen from the perspective of humanitarian actors. Despite their differences, actors belonging to the humanitarian segment share a more principled approach to policy and practical work as compared to what more pragmatic and political development actors do. The humanitarian principles of humanity, neutrality, impartiality and independence provide the foundations for humanitarian action. These principles are important in constituting humanitarian actors’ identity and legitimacy, they govern humanitarian practice and they are central in constructing the conceptual and physical humanitarian space in which humanitarian actors operate. The humanitarian principles are, however, not blueprints or a straitjacket, but propositions and values that guide action, set standards and provide benchmarks against which practice aspires and is later measured. This makes the principles subject to contextual interpretation and application, by different actors in different settings, something which affect the formation of a humanitarian–development nexus—which is the scope of this article.

First, the article outlines central policy features and challenges related to the humanitarian–development nexus. Second, these aspects are put into historical context by giving attention first to the how this nexus emerged in the wake of the need to unpack the civilian dimension of the erstwhile security–development nexus, and then to the discursive origins of humanitarianism and development respectively. The empirical third section first provides some vignette cases concerning the humanitarian–development nexus at the top-levels of policy and planning, before a more bottom-up approach is given. This section explores the discursive transition from humanitarianism to development in Northern Uganda, which triggered different and pragmatic interpretations of the humanitarian principles. The cases demonstrate how various analytical approaches produce different renderings and dynamics of the humanitarian–development nexus. Moreover, the two approaches demonstrate that the nexus itself is not only driven forward by headquarters’ policies but also by actors’ pragmatic stance vis-à-vis the humanitarian principles. Notwithstanding these different dynamics, important practical and conceptual challenges are maintained—which is the scope of the final coda section.

## Intentional policy interface of humanitarianism and development

The humanitarian–development nexus received renewed, global attention with the World Humanitarian Summit in 2016. In recognising that humanitarian assistance alone is insufficient to adequately address the needs of the world’s most vulnerable, this UN summit called for new humanitarian approaches transcending the humanitarian realm: first, humanitarians were now to engage in conflict prevention and address its root causes, which are activities not only typically designated the development segment but also activities taking place *before* the humanitarian crisis occur, thus infringing on the notion of the humanitarian present. Second, it called for increased humanitarian emphasis on political diplomacy and conflict resolution, being typical peacebuilding activities and infringing on humanitarianism’s apolitical principles. Third, and corollary, this requires bringing together humanitarian, development and peacebuilding efforts, into what got known as the triple nexus, to harmonise different, diverging and potential conflicting actors, activities and objectives. Notwithstanding the wide acclamation of the humanitarian summit—attended by more than 9000 representatives of states, governments, civil society, private sector, international and non-governmental organisations—the nexus remains widely debated, poorly adopted and highly contested. To illustrate, whilst revising this manuscript I attended two webinars concerning the nexus^2^. Bringing together practitioners and researchers, the seminars revealed a form of structural flagellantism when dealing with the challenging scope and practices pertaining to the nexus: uncertain policies, conflicting mandates and lack of leadership; limited funding, competition over resources and siloed funding streams; need to collaborate and coordinate more. This article, however, moves beyond these immediate practical concerns to engage the more discursive and institutional aspects relating to the humanitarian–development nexus as primarily seen from the humanitarian side, which illustrates how the nexus both drives and reflects changes in the humanitarian realm by expanding and contracting the humanitarian space.

The bold ambitions of the humanitarian–development nexus demonstrate that the instituted order of humanitarianism is both changing and challenged. Shifting circumstances in the areas where humanitarian actors operate and the responses these call for demonstrate how external and internal factors affect the domain of humanitarianism. This also point to an anachronism—humanitarianism’s governing principles were conceived of in a time and in response to concerns different to contemporary ones: current environmental concerns and climate change affect conflict patters; armed conflicts increasingly take place within and not between states, which makes it tricky to manage host state’s consent; not only are civilians increasingly targeted but state actors may also be amongst the adversaries; geopolitical shifts affect conflict formations and humanitarian practice; external funding agencies push for more inter-agency cooperation spanning the civil–military divide affects humanitarian practice. Despite this humanitarian anachronism between old principles and new concerns, the principles remain important in constructing the conceptual and physical humanitarian space in which humanitarian actors operate and which provides for the identity, protection and legitimacy of humanitarian actors and action. Yet, as these new and external factors impinge on the realisation of the humanitarian principles, it is regularly argued that the humanitarian ethos and rationale are challenged, that the humanitarian space is shrinking (Brassard-Boudreau and Hubert 2010).

This article, however, draws attention to how changes internal to the humanitarian domain work in the opposite direction but with the same effects, that is, the humanitarian scope is intentionally expanding, which causes a depletion of its principles and space. The formation of the humanitarian–development nexus both illustrates and propels these processes. They demonstrate that humanitarianism is intentionally changing from within, reorienting itself beyond the humanitarian present and its apolitical fundaments. These processes reflect what Barnett (2011) called the ‘humanitarian mission creep’: as the seminal humanitarian scope and ethos as constituted by the humanitarian principles are put under pressure and stretched, the conceptual and physical humanitarian space in which humanitarian actors operate are gradually truncated. For instance, the notion of the humanitarian present—i.e. the idea that humanitarian action is about the here and now, not what occurs before or after the crisis—is being undermined by humanitarian involvement in prevention and reconstruction activities. These are activities which temporality occurs before and after the humanitarian present and which traditionally has been the scope of development aid—but ‘as humanitarians began imagining how to build peace after [or before] war, they slipped into building states’ (ibid.: 3), which undeniably verges on politics and thus has a poor fit with the apolitical humanitarian principles.

Greater cooperation across segments drive the mission creep and thus changes to the instituted order of humanitarianism and how the principles, and thus legitimacy, of humanitarian action, is challenged. This calls forth institutional boundary work, where the humanitarian segment not only needs to wrestle with the security segment, but increasingly with other civilian components of the international system. The humanitarian mission creep thus reflects the ongoing nexus between humanitarian action and development aid. The encounter of these knowledge fields constitute what Long describes as situations of interface, defined as ‘a critical point of intersection or linkage between different social systems, fields or levels of social order where structural discontinuities, based upon differences of normative value and social interests, are more likely to be found’ (Long 1989: 1–2). The notion of interface implies a shift in analytical focus from different discourses or systems of knowledge towards the various situations where these meet and where differences become articulated, and draws attention to various forms of battlefields of knowledge (Hilhorst and Jansen 2010; Lie 2012; Long and Long 1992). The two discursive fields of development and humanitarianism represent two such distinct knowledge and practice fields of the international system—yet, they are invariably treated as one common, civilian segment of the international apparatus, particularly when contrasted with the military segment.

## Unpacking the civilian dimension of the security–development nexus

The means and mechanisms of the international system are traditionally described as and compartmentalised into the three distinct segments of defence, diplomacy and development. Defence or security, and diplomacy are seen to trump other concerns by virtue of protecting and promoting states’ national interests. Conversely, the development segment aims, at least nominally, to assist in promoting the interests of others, i.e. the aid recipients. There is thus an innate hierarchy between these segments, where the soft power of development is dwarfed by the hard power and realpolitik of the other segments (Leira 2019; McNeish and Lie 2010). Since 9/11 2001, however, these three segments have become increasingly and intentionally intertwined, merged into 3D or whole-of-government approaches. This merger both drives and reflects how donor countries’ national interests impact international development in general and how the politics of western aid is being securitised, especially in areas considered conflict hot-spots (Fisher and Anderson 2015). The instrumentalization of western aid has not only affected development aid’s core purpose of fighting global poverty but also collapsed the practical and conceptual distinction between security and development (Woods 2005).

The international experience with the wars in Iraq and Afghanistan from the early 2000s and the international intervention in Darfur were seminal in advancing the security–development nexus (Buur et al., 2007; Chandler 2007; Contessi 2010). Here, policymakers and security actors included the civilian development component into military operations in order to win the local populations’ hearts and minds (Gusterson 2011; González 2010). Whereas the diplomacy and defence segments represent different state entities with established protocols or rules of engagements, the development segment is often understood as more diverse and unruly by virtue of consisting of a multitude of civilian, non-state actors having their distinct mandate. Yet, those orchestrating the security–development nexus often fail to see these differences and thus largely treat the civilian component as one homogenous entity, thus undermining important internal differences amongst actors and, notably, between humanitarian action and development assistance (Carothers and de Gramont 2013). The development segment includes bi- and multilateral actors in addition to the vast NGO-sector. Despite most of these actors are in a triple bind in the sense that they receive policy guidelines from their funding agency, have their own institutional scope and mandate to attend to whilst also need to align their policies with those of their recipient. Aid actors are, however, fairly autonomous in brokering and juxtaposing these potentially conflicting interests. Understanding these dynamics are crucial not only to understand the development segment itself but also to grasp the problems and opportunities that lie in the interface between various actors and segments of the international system. Seeing the civilian component of the international apparatus as a homogenous development segment not only reduces the complexity of civilian engagement. It also undermines the distinct nature and role of its two main sectors of development assistance and humanitarian action, and the important differences that exist between them. Whilst these differences may appear opaque from an outsiders’ perspective, they are central to the individual sectors’ identity, legitimacy and boundary work vis-à-vis other institutions.

Despite these differences, calls are being made for greater coherence, cooperation and harmonisation *within* the civilian segment. Whereas the security–development nexus sought to combine military and civilian efforts, these calls proposed a new way to manage the complex war-to-peace transition by proposing a new workflow between civilian actors in what would be identified as a humanitarian–development nexus (Acuto 2014; Lie 2017; Yamashita 2015; Eade and Vaux 2007; Chandler 2014; Barnett and Weiss 2008). This nexus is driven forward by not only external impulses and political intentions, but equally by changes internal to the segment itself and its immanent responses to new conflict formations and humanitarian concerns. Understanding the war-to-peace transition as representing a continuum and not distinct and sequenced phases designated military, humanitarian and development actors has been pivotal to foster this new nexus (Duffield 2012). The nexus fosters the intentional, but challenging interface of humanitarian action and development assistance, and it brings humanitarianism into the development domain of prevention and reconstruction. Consequently, it widens the humanitarian space through a more contextual and pragmatic application of the humanitarian principles. As the nexus touches on the very identity and leitmotif of both humanitarianism and development, these interfaces require a great deal of boundary work between erstwhile distinct segments of the international system. However, to explore these interfaces one first needs to understand the segments.

## Humanitarianism and development

The fields of humanitarian action and development aid represent two different segments of the international system with distinct discursive origins and institutional trajectories (Hilhorst 2018; Dudaite 2018). The origin of humanitarianism typically pivots around Henry Dunant and his experience at the Battle of Solverino in 1859. Originally on a business travel, Dunant accidentally witnessed a carnage between Austro-Hungarian and French troops outside the Italian village of Solferino, which made him join the local townspeople to provide the relief he could. Dunant had ‘left Geneva as a man seeking riches and returned home as a man who was about to dedicate his life to higher calling’ (Barnett 2011: 1). After publishing his memoirs, which became a European bestseller, Dunant launched a call to alms and a campaign to establish a permanent relief agency for humanitarian aid in war, and a government treaty recognising the neutrality of this agency in order for it to operate in a war zone. Following a broad grassroots movement, this led to the establishment of the International Committee of the Red Cross (ICRC) and the Geneva Conventions, which constructed the humanitarian discourse around a set of guiding principles that still epitomise and shape humanitarian action today, despite being revised and reaffirmed in 1949 to account for the lessons of two World Wars and the establishment of the United Nations (Fassin 2012; Terry 2002). Later, these principles have become formally enshrined in two UN General Assembly resolutions. The principles of humanity (that human suffering must be addressed when and wherever it is found), neutrality (humanitarian actors must not take side) and impartiality (aid should be based on needs alone) were adopted in 1991 in a pivotal resolution marking the (re-)creation of the humanitarian system and the international community’s collective commitment to help the world’s most vulnerable when they need it most^3^. The principle of independence (that humanitarian action must be autonomous to any other concerns, such as political, military and economic objectives) was added in 2004^4^, and can be seen as a response to the incipient security–development nexus. These principles guide humanitarian action, which implies they may be subject to different interpretations and context-specific usage. They are nevertheless important for the humanitarian identity and in constituting the humanitarian space, the humanitarian present and humanitarianism as apolitical.

The institutional development apparatus has a more intentional and political origin. Emerging in the wake of the Second World War and the ensuing geopolitical struggles, aid and economic support were seen as means to establish and secure political interests. Inspired by and extending beyond the Eurocentric Marshall plan, scholars usually associate President Truman’s 1949 inaugural speech as the inception of ‘development’, marking the start of a particular historical period—the era of development (Sachs 1992). Point Four of the Truman Doctrine established lasting master metaphors in stating that ‘We must embark on a bold and new programme for making the benefits of our scientific advances and industrial progress available for the improvement and growth of underdeveloped areas’ (Porter 1995: 66). It ordered the world into ‘us’ and ‘them’ by creating a conceptual division between the ‘developed’ and ‘underdeveloped’, where the latter is defined by lacking what constitutes the former, i.e. development (Nustad 2001). This stipulates not only what development is, but also that development is something to be actively conveyed ‘them’ by ‘us’. This undercuts the idea of development as an immanent process unfolding over time to advance a perspective that development requires an active and guided societal intervention—as later manifested by the modernisation theory drawing on Rostow’s guide to unilineal progress in different qualitative stages with the USA as the beacon on the hill, showing way and calling every nation to follow in its footsteps (Cowen and Shenton 1996; Escobar 1995). Development assistance today is highly diverse, ranging from small NGOs to huge multilateral institutions working in partnership with recipient institutions no projects spanning local development and social development to good governance, economic reform, infrastructure development and state building. Development work is political both in scope and organisation and draws on a rights-based discourse. At the operational level, development aid builds on certain partnership principles where participatory approaches aim to instal recipient ownership and decisiveness to externally funded programmes. These partnership principles are, however, rarely realised in practice and donor countries still use aid for political purposes.

The different discursive origins of humanitarianism and development have produced and reproduced two distinct civilian segments of the international system that often appear to be at odds with each other: humanitarianism’s apolitical and imminent needs-based approaches building on the principles of neutrality, impartiality and independence are fundamentally different from the more long-term, political, and rights-based approaches of development. Humanitarian action is mainly exogenous, meaning that interventions, ideas and funding come from outside of the affected country, building on universal principles and often in response to the host state’s lack of will or capacity to sufficiently cater for its own citizens. Contrary, development aid is organised around certain governing partnership principles, meaning that both donor and recipient institutions—be it NGOs, state or multilateral institutions—are supposed to jointly programme and implement projects and that external actors should underpin the policies and priorities of the recipient. In such efforts, the ambition is that those on the receiving end should have ‘ownership’ to externally funded processes and policies, and the donor agencies should align their approaches and policies with those of the recipient.

The segments of humanitarianism and development differ in both structure and content regarding how aid is provided and what type of aid is given. These practices are shaped by and keep reproducing the segments’ formal distinctiveness, historical trajectories and institutional orders. Yet, against such a formalistic notion it is important to underline that each segment consists of a myriad of different actors, from civil society to multilateral organisations, operating in very different contexts, which provide different practical and pragmatic interpretations of the governing principles of humanitarian action and development aid. As such, the renderings of these segments and their governing principles are not only contextual. The principles governing humanitarian and development practices are of a more philosophical concern taking place in the conference rooms at the headquarters level (Slim 2006), whereas the imperative to help prevails in field operations regardless of principles and segments. Indeed, the beneficiaries and those at the receiving tend not to tell apart the various actors and their principles regardless whether they are described as belonging to either of the humanitarian or development domains (Lie 2017). Yet, these differences matter: both for how and what aid is provided, and for the institutional cooperation in the field and at headquarters level. The empirical cases below first attend to the problematic interface of development and humanitarianism at the headquarters level, illustrating the philosophical quandaries of implementing the humanitarian–development nexus. Moving from the empirical vignettes, the case from Northern Uganda takes a bottom-up approach to the nexus. It demonstrates the interface of the different discursive segments in practise and how the nexus is being propelled not by top-driven policy guidelines but as an effect of how actors in the field are more pragmatic about the principles to maintain relevance as the government recasts the humanitarian crisis situation as one of recovery.

## Vignette cases on the interface of development and humanitarianism

When I attended the launch of a joint UNHCR–World Bank report—*‘*Forcibly Displaced Towards a development approach supporting refugees, the internally displaced, and their hosts’—the authors, and in particular the one representing UNHCR, stressed the hardship that had been put into having two different organisations with distinct mandates speak together^5^. Amongst the rationales of these two multilateral agencies joining forces was to put together their distinct datasets, to monitor and predict migration patterns in order to intervene before people start migrating. The cooperation draws on the notion that development interventions could provide preventive measures by helping displaced and host communities before they migrate. This interface not only reflects the humanitarian–development nexus, but also how aid is being politicised and installed with other interests than held by the two segments themselves as the cooperation was arguably pushed for by donor countries’ aiming to limit migration to the global north. These political considerations never surfaced during the report’s presentation and discussion, and indeed the substance of the report itself received little attention. Rather, both the World Bank and UNHCR representatives stressed the novelty and problems in working together, reiterating their organisation’s mandate and distinctiveness and thus the interface challenges in having the various units in DC and Geneva operate together.

Similarly, when UN and World Bank staff facilitated various regional, consultative meetings in preparation of their joint flagship study ‘Making Development Work for the Prevention of Violent Conflict’ (UN and World Bank 2018)^6^, a seemingly disproportionally amount of time and focus were devoted to how one could have the UN in New York and the Washington-based World Bank speak together at the institutional level. Here, the staff said they were personally committed to this work and to working together, but again: finding a common ground and joining the mandates of the different organisations had been challenging at the institutional level and arguably amongst the more serious concerns preventing collaboration.

In early 2014, UN OCHA launched its policy initiative *Saving Lives Today and Tomorrow* (SLTT), being yet another flagship report that calls for a fundamental shift in the way humanitarian and development actors operate^7^. OCHA, itself a gatekeeper of the humanitarian principles, initiated the report and policy initiative. As the title indicates, the report calls for an expansion of the humanitarian present into future crises through preventive measures to also save lives tomorrow. The policy initiative calls upon the funding agencies to shift from responding to crises in a purely reactive manner to instead adopt a proactive approach to anticipate and prevent crisis through risk management initiatives. Realising that existing structures are not sufficient, the SLTT policy initiative seeks to overcome institutional hurdles to foster greater cooperation between different actors to invest in risk mitigation and crisis management. The SLLT policy states that to ‘overcome this challenge, humanitarian, development and government actors must work together to identify risks and align planning cycles, increase aid effectiveness, build the resilience of affected populations and, where possible, focus on preventing disasters’^8^. As reported by several UN and World Bank, this initiative fast gained momentum outside OCHA. Representatives of other UN entities—such as DPKO, UNDP and DPA—all held that they saw this as a sign that OCHA was opening up its humanitarian gatekeeping role and what they saw as a strict interpretation of the humanitarian principles. Turbulence soon surfaced within OCHA as not all were convinced with this policy initiative and the problems bound to occur when ‘aligning planning cycles’ with complex development actors external (World Bank) and internal to the UN (UNDP). OCHA was, moreover, concerned how both the UN entities of DPKO and DPA also got on board the SLTT policy initiative, thus sparking critique within the humanitarian segment about aligning its cycles not only with UN’s political affairs unit but also its military arm (i.e. DPKO). Many in OCHA saw this as a bridge too far, reportedly causing OCHA to effectively (but not nominally) withdraw its support for the SLTT-initiative to the extent that all practical momentum was lost.

The humanitarian discourse may also be adopted by those external to it, as a means to gain legitimacy to own operations. When the DPKO from 2009 incepted its New Horizon-initiative to chart out new roles and functions of UN peacekeeping operations^9^, it included the Protection of Civilians (PoC) as an integral and central part to future peacekeeping operations (Lie and de Carvalho 2009). The reference to PoC would no longer only be in the mandate, with the many uncertainties that entailed when translated into practice (de Carvalho and Lie 2011). A strategic challenge to peacekeeping operations as identified and to be ameliorated by the New Horizons-agenda, was how modern peacekeeping struggles to come up against the reality of insufficient instruments to address political issues in the areas of operations. This concern was propelled due to two interconnected facts. Firstly, that conflicts and peacekeeping operations now take place within states and not between them, which was the case when the DPKO was formed. Secondly, the mandate of peacekeeping operations needs to be negotiated and approved by the host government, which tends to refute any references made to politics, thus tying the hands of DPKO and DPA to work politically with what they regard as the root causes of the conflict. Here, the humanitarian discourse and, in particular, the domain of PoC served as a Trojan horse to work politically through a seemingly apolitical mission mandate. As the mandate of UN missions merely refers to PoC as ‘to protect civilians under imminent threats of physical violence’, without further telling what this actually entailed, it became up to the force commander or others within specific missions to interpret the PoC agenda and put it to action. So, whilst PoC up until the New Horizons agenda was merely seen as a bothersome humanitarian mandate requirement, it now gained new momentum—although not always for the reasons initially envisaged by the humanitarian proponents.

The vignette cases illustrate different expressions of the humanitarian mission creep, driven forward by bold policy headquarters ambitions pertaining to the humanitarian–development nexus. The cases render the different interfaces between the discursive realms of humanitarian action and development aid, illustrating contentious knowledge battles at the institutional and policy levels that also may also undermine the headquarters’ policy ambitions. These interfaces bring together different principles and institutionalised mandates, demonstrating how the nexus itself is both produced by and contested at the top. Below, however, a more ground-up perspective is offered to the formation of the humanitarian–development nexus. Drawing on fieldwork exploring civilian protection in Northern Uganda (Lie 2012, 2017), the case offers a different perspective to the nexus formation. The case illustrates how the mission creep and nexus are driven forward not by central policies but as a result of changing crisis context and humanitarian actors’ pragmatic use of the principles when operating in a volatile environment. Indeed, by the stroke of the pen, the Ugandan government recasted the situation in Northern Uganda from being one of crisis and thus the scope of humanitarian action, into one of recovery, which is the domain of development aid. Redefining the situation as one of recovery produced obstacles to humanitarian actors and their ability to pay heed to the humanitarian principles. Whereas some withdrew, others took a more pragmatic stance to this recast and the humanitarian principles under the auspice of the imperative to help the civilians regardless of how the situation was being (re)defined.

## Northern Uganda: conflict to recovery - humanitarianism to development?

In 2005, at the peak of the civil war between the Government of Uganda and the Lord’s Resistance Army (LRA) there were roughly 1.8 million internally displaced people (IDPs) living in 251 different ‘protected camps’ across 11 districts in Northern Uganda. The roots of the conflict dates back to 1986 when current President Yoweri Museveni and his National Resistance Army (MRA) overthrew former president Tito Okello, who came from the northern Acholi tribe. Museveni came to power promising to restore stability, security and respect for human rights. People from the northern parts were, however, antagonistic to a government led by a person from the south. Museveni’s policies of downplaying ethnicity as a political, organising principle were seen as a way to undermine the political voices of the north, creating further trepidation in the north, causing rebel groups to emerge. Multiple insurgencies emerged from Acholiland, of which the Joseph Kony-led LRA would eventually rise as the most enduring and destructive rebel movement.

In response, the government in 1996 escalated its fight against the LRA through its scorched-earth policy. This included ordering all Acholis to vacate their homes in 48 h, forcing them to resettle in ‘protected camps’, thus swiftly turning the citizens into internally displaced people (IDPs). The rationale was to separate civilians and combatants in order for the Ugandan People’s Defence Force (UPDF) to identify and target the combatants. The government invoked a humanitarian reasoning for the encampment process, arguing the camps would enable the UPDF to better offer physical protection. Seeing the political aspects of the insurgency, however, allows analysing the encampment and recasting the civilians as IDPs deprived of their civil rights as a way to control and quell political opposition from arising in the north, thus being instrumental in the Kampala-based state formation process.

The so-called ‘protected camps’ did not receive sufficient physical protection as the UPDF used its limited resources to focus on fighting the LRA and not protecting the civilians. This left the camp borders porous, allowing the LRA to hide and attack within the camps. The civilians, moreover, were forced to move outside the camps to maintain agricultural production, collect firewood, go to the markets and so on, as there was a critical lack of services and provisions within the camp. Leaving the camps to sustain their livelihood, the civilians feared not only being attacked by the LRA but also being associated with the LRA and consequently reprimanded by the UPDP since the government’s policy criminalised out-of-camp activities. Together, the LRA atrocities, the UPDP’s response to the LRA and the government’s encampment policies and poor camp management produced a massive humanitarian crisis (Branch 2011).

International humanitarian assistance was gradually phased in to help respond to the ensuing humanitarian crisis. In response, the government reallocated its camp funding into the UPDF, making the humanitarian involvement at least partly complicit in sustaining the conflict as it allowed the government to focus on a military and not political solution to the conflict, whilst international humanitarian actors assumed responsibility for managing the camps. Meanwhile, most state building and development activities in the north were lying fallow with neither funding nor focus from the government and international actors.

Northern Uganda experienced a dramatic expansion of externally funded, humanitarian civil society organisations from the late 1990. The intervening humanitarian regime had its primary function in administrating camp populations, causing civilians to be self-disciplined into non-political forms of organisation rather than empowered to pursue their own aspiration. The massive influx of humanitarian actors proliferated after the head of UN OCHA, Jan Egeland, in 2003, brought attention to the conflict, describing it as the worst forgotten humanitarian crisis on earth, followed by pledges and appeals to beef up relief operations. The ensuing CNN-effect made humanitarian funding skyrocket, from $34 million in 2002 to its 2008 peak of $238 million, causing Northern Uganda, in the words of an informant, to suffer from NGO-obesity, creating an aid-based civil society and an economy almost entirely contingent on external funding.

The peak of the external involvement in 2008 also marked the beginning of the end of the humanitarian crisis—at least nominally—as the government started a process of recasting the situation from being one of humanitarian crisis to one of recovery and development in order to reclaim its sovereignty in Northern Uganda. Instrumental in this move was the government’s closure of the ‘protected camps’, thus forcing the internally displaced population to return to their ancestral fields which had been lying fallow for over a decade. The government’s discursive recast of the situation happened partly in response to how the humanitarian actors undermined the state through their comprehensive operations and antagonising advocacy work, and partly because the UPDF had pushed the LRA out of Uganda. The government took the Kampala-based donors to the north, showing them that people were returning home, that the camps were being closed and that the conflict was over as the LRA had been pushed into the neighbouring countries. Only seeing the surface of the situation, the donors were persuaded that the crisis was over and to move their funding from humanitarian activities into development aid, notably in the form of budget support to the government’s own recovery and reconstruction plans in the north.

The transition happened in spite of the persistent humanitarian sufferings and needs in the post-conflict period. The recast had detrimental effects for ongoing activities—‘too many NGOs withdrew too soon with too much unfinished business’ was the general story told by informants, arguably leaving a humanitarian vacuum for the many civilians who after years in the protected camps were forced to leave, returning to their homelands and districts which in the meantime had received minimal government and donor attention regarding social and infrastructure development.

Admittedly, representatives of local authorities, humanitarian and development NGOs and community leaders all expressed that there no longer was an ongoing crisis per se given that the armed conflict had ended, which warranted a move into recovery and development. Yet, they held there were numerous concerns with the immediate camp closure and the depleting humanitarian funding. Although the LRA had been pushed out of Uganda, it still waged attacks but with plummeting frequency from neighbouring DRC and South Sudan.

The sudden decommissioning of the IDP-camps undermined the sustainability of the humanitarian’s work. The process of replacement was, moreover, full of tensions as the IDPs themselves were never consulted about their repatriation as stated by the principles of Durable Solutions for IDPs. Formally, the government subscribed to these principles. In practice, however, the authorities wanted the displaced people to return to their rural homes as quickly as possible, arguably as a way to reclaim its humanitarian sovereignty and as a token of stability and the transition to recovery.

The returning IDPs, however, were faced with a collapsed state apparatus that had received little attention from both donors and the central state during the decades of armed conflict, during which the many NGOs in effect had replaced the state in terms of service delivery and protection efforts. The humanitarian activities during the crisis had, moreover, addressed the immediate and present concerns, but in so doing the intervening actors neglected foreseeing the future, post-conflict needs and concerns. Land rights, in particular, became a critical hotspot causing for violent conflict. Many families saw their land they were forced to move from appropriated when returning from the camps, causing for legal disputes erupting into violent conflict due to the lack of formalised tenure and a penal system to settle the conflicts.

The camp closure not only degraded the livelihood fundament and basic services provided by the humanitarian actors. The ensuing forced resettlement also dismantled the social fabric that had been established within the camps. Together, this exacerbated the humanitarian concerns and needs in the post-camp, recovery phase despite the conflict had formally been called off. These concerns were further exacerbated as the humanitarian actors started to phase out after losing their rationale and operational consent when the government did recast the crisis situation as one of recovery. In the areas recovering from decades of armed conflict, civil society and local authorities reported about concerns related to children’s rights, sexual and gender-based violence, poverty, unemployment, food insecurity, violence, conflict over access and rights to land, unexploded ordinances and weapons, illiteracy and a general lack of basic services such as health, social protection, education and water.

### Responding to the transition—principles and pragmatics

Are these concerns of a humanitarian or development character? Within which realm do they Uganda. Indeed, perhaps with the exemption of the contentious land rights, all other issues are found throughout the country where they are addressed by development actors. The main difference, however, is the magnitude and complexity of these issues caused by decades of conflict. These issues, moreover, became exacerbated due to limited attention given to infrastructure development and state building during the conflict, together giving these concerns both a temporal and spatial diffusion outreaching any other national context.

Generally, the humanitarian actors’ response to the government’s situation recast can be grouped into three categories: those who withdrew with reference to their humanitarian mandate; those who relocated their operations and those who maintained presence but refocusing and reorganising their aid activities. Of the first category, one finds the UNHCR, the ICRC and the MSF. The UNHCR had to withdraw as it is dependent on both the government’s consent and donor funding to operate. The ICRC and MSF—being actors that well beyond this specific case are renowned for their strict and verbatim understanding of the humanitarian principles—withdrew with reference to their humanitarian mandate as the situation moved into recovery and development. They acknowledged the critical situation and the persistent needs but feared breaching humanitarian principles, explicitly invoking a hierarchy by holding humanitarian principles over pragmatic approaches. The second category involved several larger, international NGOs that sought to pay heed to the humanitarian principles whilst simultaneously remaining operational in Uganda due to funding requirements. This was enabled by relocating operations to the north-eastern Karamoja-region where the indigenous pastoralists’ cattle raiding dynamics, climate change, land scarcity and nomadic transborder movement now emerged as a humanitarian hotspot requiring intervention. The third category involves organisations that took a more pragmatic stance to the discursive recast of the situation in the north and its consent and funding implications. Disagreeing about the underpinning assessment and recognising the persistent concerns, some actors reframed their programmes into development aid, which warranted both operational legitimacy and external, financial support. Despite scaling down their activities due to the plummeting of funds, these organisations gradually aligned themselves with regular development activities, such as building schools, education, reproductive health, vocational and livelihood training, agricultural extension programmes and reintegration projects.

These organisations, in disagreeing about the government’s assessment to warrant the discursive recast of the situation, took a more pragmatic stance regarding the humanitarian principles. Humanitarian actors that took a more pragmatic stance, argued that the government’s rhetorical recast of the situation was unsubstantiated, did not reflect real changes on the ground and was motivated by having external organisations to withdraw. As argued above, the discursive recast did not eliminate humanitarian concerns. Some were even exacerbated when people had to migrate as the camps closed. Moreover, the organisations were concerned that their donors did not realise the persistent humanitarian needs and that the resettlement of civilians, which persuaded donors about the discursive recast, in fact was a forced one caused by the government’s closure of the camps. Against this backdrop, several organisations maintained their operational activities, arguing that their commitment to the civilians and those suffering in the post-conflict period trumps the government’s rendering of the situation as well as the sanctity of the humanitarian principles. ‘We are committed to helping people in need, not to maintain some abstract principles’, one informant held. Reflecting similar sentiments, several organisations reoriented and redefined their support, moving from explicit humanitarian assistance into development aid ‘but with a significant humanitarian touch’, I was told. For instance, the Norwegian Refugee Council, despite significantly scaling down its activities, moved into infrastructure development by building schools. For humanitarian purposes, this was inscribed as part of the education in emergencies discourse, although there was no emergency and even those doing the work perceived it as a typical development activity (Lie 2017). Initiatives from, amongst others, the Justice and Reconciliation Project, Save the Children, and Refugee Law Project all received less humanitarian funding, yet they aspired to maintain their activities with reference to persistent needs. Whilst some of these organisations are so-called dual-mandate organisations, meaning they attend to both humanitarian and development situations, their funding streams and operational consent are usually tied to either of the segments.

To transcend segments involved boundary work, downplaying the unilateral relief and advocacy work based on the humanitarian principles, to advance greater partnership with the local communities and their participation in, and ownership of, development policies that were also required to be aligned with the government’s national development strategy. This meant that policies and activities nominally had to conform to those of the government, such as infrastructure capacity building, education, health services, vocational and livelihood training and farming activities. When recasting their work in terms of development, less attention was given to issues of human rights, gender-based violence, child protection, legal aid and reproductive health, although most organisations started to address these issues indirectly and as part of less controversial topics.

Throughout, informants held that the transition from humanitarianism to recovery happened too fast, and that neither donors nor implementing agencies were sufficiently prepared for this shift. Their work and strategies were planned for emergency responses and humanitarian funding, not development and rehabilitation. ‘Those organisations that did not alter their practices according to the new rendering of the situation would eventually have to withdraw’, one informant stated, adding that perhaps the most critical aspect was the lack of an exit strategy to ease the transition, to prevent abandoning a lot of incomplete work, and mitigate the impact the sudden transition would have for the beneficiaries. Consequently, against this backdrop and the persistent humanitarian issues still lingering in the area, a number of organisations reoriented their support as development, both as a way to ease the transition, complete ongoing work, and phase out activities in a somewhat more sustainable manner, but also as a way to continue address the persistent humanitarian needs.

This pragmatic stance in transitioning from humanitarianism to development was less controversial to the donor community and the national authorities than to the humanitarian agencies. The donors admittedly struggled to find new partners to receive the funding already allocated to their budget for activities in Uganda. National and local authorities were satisfied, not only because the humanitarian crisis was formally over but also that donors would commit themselves financially to assist in the government’s state-building and recovery activities now planned to take place in the north. The organisations that withdrew or moved into development activities were antagonistic as they did not see the transition being warranted by any substantial improvements of the civilian’s needs—yet they were compelled to relate to the transition, although in different ways.

Amongst those organisations withdrawing according to their humanitarian mandate, this pragmatic ‘third way’ response was seen as somewhat controversial and a hollowing of the humanitarian space and principles, as it connected erstwhile humanitarian actors not only to the politics of aid but also to the government’s state building and recovery programme. In the words of an informant responding to this critique, ‘we are committed to help people in need, not to maintain some abstract principles’. The different responses to the discursively recasted situation remind us not only about the heterogeneity of humanitarianism but also the malleability and its constitutive principles: multiple and diverse organisations operate under the same humanitarian umbrella and lend legitimacy from its morally charged principles and values, regardless whether organisations share the same interpretation and understanding of what these principles mean and entail in practice. The malleability of the humanitarian principles and concepts make for a knowledge battlefield where different actors representing different organisational cultures and mandates vie over humanitarianism’s meaning, interpretation and application in practice. This malleability is to some seen as a way to be pragmatic about humanitarian challenges and principles by enabling more diverse and context sensitive operational action—meaning that the end justifies the mean. Conversely, others see the malleability as undermining the humanitarian principles and the legitimacy they provide for, thus curtailing room to manoeuvre on the basis of humanitarianism.

## Coda: The compartmentalisation of humanitarianism and development

The discourses of humanitarian action and development aid are often understood as representing different archetypes (Ferme 2013), megarhetorics (Dingo and Scott 2012) or communities of international aid (DeChaine 2005). Increasingly, these segments of the international system rub shoulders—as tectonic plates—affecting, overlapping and challenging each other. Their interface illustrates an incipient humanitarian–development nexus. Regardless whether this nexus is produced by intention or as an effect of how actors pragmatically employ the humanitarian principles in practice—as illustrated by the vignette cases and the Northern Uganda case respectively—it nevertheless reflects an ongoing humanitarian mission creep (Barnett 2005, 2011). This may potentially undermine the humanitarian principles and thus the humanitarian space and legitimacy so central to the conceptual and physical humanitarian practice in which humanitarian actors operate (Acuto 2014). In its strictest sense, humanitarian action is understood as the unconditional help to people in need, when in need. Corollary, when needy people are not offered assistance or if assistance is provided before needs arise, this nominal version is undermined. As the cases illustrate, this nominal version is being stretched both through practices emerging from the bottom and by top-driven policy initiatives: practitioners increasingly engage aspects outside of the humanitarian present, see the war-to-peace transition as a continuum, and thus decompartmentalise how various actors operate in distinct (temporal) phases of the crisis. This, however, reflects back on the seminal humanitarian scope. To return to Barnett’s quote in the beginning: ‘as humanitarians began imagining how to build peace after war, they slipped into building states’ (Barnett 2011: 3), being not only the domain of development but also an inherently political activity taking place before needs arise. The obvious question that begs itself is whether this really matter—should principles or pragmatics prevail and guide action? Or is this conceptual and philosophical concerns merely relevant in the conference rooms and at the headquarters levels? Do the humanitarian ethics become tainted when humanitarian agencies take on practices outside its remit? Is there a hierarchy, or contradiction, between the humanitarian deontology and pragmatic approaches to save lives and protect civilians?

The cases above show different dynamics and considerations regarding these questions. The vignette cases all demonstrate the troublesome practical interface of the two distinct discourses of humanitarianism and development at the level of planning, despite actors’ own policy ambitions of enhancing the nexus. This discrepancy between policy and practice is largely due to the discursive formation of the two segments and the governing role of the humanitarian principles. However, the formative role of the humanitarian principles appears somewhat different when approached analytically bottom up. As the case from Northern Uganda shows the principles serve to anchor, not govern, practice. In response to the discursive recast of the crisis, different humanitarian organisations interpret their mandate and response differently, thus showing the principle’s context-specific malleability. The transition from humanitarian action to development aid in Northern Uganda illustrates the ambiguities of principles and pragmatics by drawing attention to important humanitarian changes and challenges that give momentum to what has been described as an ongoing humanitarian mission creep causing for a general debate about the nature and future of humanitarianism. As the case demonstrates, this mission creep is driven by interrelated factors both external and internal to the humanitarian field. Externally, the recast of the situation into one of recovery impinged on the government’s consent and donors’ funding of the humanitarian activities. Internally, realising the lingering and persistent effects of the Civil War several humanitarian organisations reoriented their support in terms of development in order to continue to assist the civilian population, thus taking a pragmatic stance favouring the civilians’ needs and not the humanitarian principles.

The case of the humanitarian changes and challenges in Northern Uganda correspond to more general observations pertaining to the evolving humanitarian mission creep, i.e. that the humanitarian–development nexus is driven not only by central policy prescriptions but also by how actors in pragmatically and in practice respond to changing and complex contexts demanding new approaches (Duffield 2007; Kaldor 2007; Terry 2002). In response to these changing circumstances and new conflict formations, humanitarian actors have expanded their scope beyond their seminal temporal and conceptual remit so important for the humanitarian principles and thus actors’ legitimacy. On the temporal side, it questions humanitarianism’s inherent presentist stance (Reid-Henry 2013; Weizman 2012), that is, the insistence on assisting people in need *when* in need. The increased humanitarian focus on prevention and reconstruction is not only commonly seen as falling within the temporal scope of development but also overlapping its thematic area of operations.

Whilst beneficiaries of international aid tend to be neglectful or unaware of the differences between humanitarianism and development aid, their distinctiveness matter for practitioners and operational activities. Increasingly these segments of the international system rub shoulders, sometimes even overlapping and challenging each other. The realms of humanitarianism and development draw on distinct rationales involving different actors with their particular mandates: humanitarianism’s imminent needs-based approaches building on the principles of neutrality, impartiality and independence—together providing for the physical and conceptual ‘humanitarian space’ in which humanitarian actors operate—are as such fundamentally different from the more long-term, political, rights-based development approaches. Central here is the notion of politics. There is no novelty in stating that both humanitarian and development aid produce political *effects* (Yamashita 2015). What we see now, however, is that political intent, motives and actors increasingly become part in shaping and defining the apolitical humanitarian realm in practice.

Humanitarianism and development may appear alike to those outside their institutional boundaries. The beneficiaries of international aid are not concerned with the institutional boundaries between humanitarianism and development. But who and how aid is delivered have important bearings and effects for the beneficiaries. Yet, and nominally, humanitarianism and development are distinct sectors in many ways, involving particular rationales, actors funding mechanisms and, importantly, their relationship to both politics and the host government in the country which they operate. Humanitarian practice is based on an apolitical and impartial rationale, often bypassing the state. Development aid is explicitly political and works with and through state authorities. These organisational and institutional differences have effects, creating great philosophical disputes in the conference rooms about the nature and future of both development and humanitarianism. In practice, however, lines are a bit more blurred. The typical humanitarian practice was concerned with providing food and blankets, which has expanded into managing refugee camps, food distribution, provide clean water and offer public health care. Other recent but less typical humanitarian practices now include ‘cash transfers, protection programming, shelter, family reunification, agricultural recovery, business continuity and humanitarian advocacy’ (Slim 2015: 13–14). Such topics overlap and replicate much of what the development sector had been alone in addressing for a long time, thus illustrating the humanitarian mission creep by moving attention from what Arendt called *zoë* to *bios*. Zoë refers to the zoological life, or the simple fact of biological life. Bios, by contrast, refers to the biographical life: ‘life that is properly formed through events such that it can be narrated as a story’ (Redfield 2005: 340). This expansion of the humanitarian scope into new forms of relief concerned with the biographical life and societal resilience has led some critical theorists drawing on the works of Foucault and Agamben to analyse humanitarianism as a form of bio-political governance (Agamben 2005; Foucault 1991). Whilst this is a common approach to and critique of the institutional development apparatus, seeing humanitarianism as a powerful instrument and a way to govern individual bodies is thus also, indirectly, a token of changes to the humanitarian field and its nexus with development.

## Data Availability

Not applicable

## Footnotes

1 I am indebted to Hannah Dönges, Benjamin de Carvalho and the two anonymous reviewers for their comments and feedback on earlier versions of this article. This research was supported by the Research Council of Norway via NUPI’s SIS Research Program (grant no. 304516), and the projects ‘Protection of Civilians: from principle to practice’ and ‘Developmentality and the anthropology of partnership’ (grant no. 262524).

2 One was the “Triple nexus in practice – what about peace?” organised by the Centre for Humanitarian Action 26-27 October 2020 – see https://www.chaberlin.org/en/event/cha-conference-on-the-triple-nexus-october-2020. The other was “Make or break: the implications of Covid-19 for crisis financing”, organised by International Association of Professionals in Humanitarian Action and Protection (PHAP) 15. September 2020 – see https://phap.org/PHAP/Events/OEV2020/OEV200915.aspx

3 See Resolution 46/182 adopted by the UN General Assembly on 19 December 1991.

4 See Resolution 58/114 adopted by the UN General Assembly on 17 December 2003.

5 Report launch at NORAD (Norwegian Agency for Development Cooperation), 26 September 2016.

6 Meeting: “Nordic Consultation on United Nations and World Bank study on development and conflict prevention: open meeting” at PRIO in Oslo, jointly organised by the UNDP’s Oslo Governance Centre and and the Peace Research Institute Oslo (PRIO), 20 October 2016.

7 https://www.unocha.org/sites/unocha/files/OCHA%20SLTT%20Web%20Final%20Single.PDF

8 https://www.unocha.org/legacy/what-we-do/policy/resources/saving-lives-today-and-tomorrow

9 http://www.un.org/en/peacekeeping/documents/newhorizon.pdf
